# Traditional Food Systems as Nutrient Optimization Architectures: Mechanisms of Bioavailability and Dietary Resilience

**DOI:** 10.3390/nu18091448

**Published:** 2026-04-30

**Authors:** Corina-Aurelia Zugravu, Marta Petre, Ciprian Constantin

**Affiliations:** 1 Department of Hygiene and Nutrition, “Carol Davila” University of Medicine and Pharmacy, 050463 Bucharest, Romania; 2 National Institute of Public Health, Dr. Leonte Street 1-3, 050463 Bucharest, Romania; marta.petre@insp.gov.ro; 3 Research Metabolism Center, Faculty of Medicine, Titu Maiorescu University, 1, 76 Al I Cuza Blvd, 011053 Bucharest, Romania; ciprian_constantin@yahoo.com; 4 “Carol Davila” Emergency Hospital, 1, 88 M. Vulcanescu Street, 010825 Bucharest, Romania

**Keywords:** traditional food systems, nutrient bioavailability, food structure, fermentation, food pairing, nutrient utilization, dietary resilience

## Abstract

Traditional food systems have historically sustained nutrient adequacy under conditions of environmental variability and limited food diversity, yet their underlying nutritional mechanisms remain insufficiently integrated into contemporary nutrition science. This article attempts to provide a conceptual synthesis of how traditional dietary practices may function as informal nutrient optimization strategies. Drawing on evidence from nutrition science, food chemistry, and human physiology, it examines how food processing techniques (e.g., fermentation, soaking, germination, and thermal treatment), food pairing, and structural properties of foods influence nutrient bioavailability, absorption, and metabolic responses. Across diverse dietary contexts—including Mediterranean, agrarian cereal–legume, and East Asian-type patterns—recurring mechanisms emerge that can boost mineral solubility, improve protein digestibility and amino acid balance, facilitate vitamin absorption, and modulate glycemic responses. These effects are mediated not only by nutrient content but by interactions within the food structure and at the meal level. The synthesis supports a reframing of traditional diets as functional nutritional architectures in which processing and dietary configuration may enhance nutrient utilization efficiency. From this perspective, nutrient adequacy arises from coordinated structural features rather than from maximal nutrient density alone. The findings can be influential in contemporary nutrition research and policy, highlighting the need to move beyond reductionist intake-based models toward integrated approaches that account for bioavailability, metabolic handling, and dietary context. Several transferable principles of nutrient optimization are proposed, offering a framework for designing nutritionally efficient and resilient diets in modern settings.

## 1. Introduction

Despite major advances in food production and nutritional knowledge, micronutrient inadequacies and diet-related metabolic disorders remain highly prevalent worldwide [1,2]. Contemporary nutrition strategies have largely relied on nutrient-specific interventions, such as fortification, supplementation, and reformulation, to address these challenges. While effective in targeted settings, such approaches often neglect the complexity of food matrices, nutrient–nutrient interactions, and the physiological processes that govern nutrient bioavailability and metabolic utilization [3].

Historically, human diets developed under conditions of environmental variability, seasonal scarcity, and limited technological capacity for food preservation. Within these constraints, food systems evolved and supported nutritional adequacy through processing practices rather than through external nutrient inputs [4,5,6]. The nutritional coherence of traditional food supply chains likely reflects a long process of empirical trial and error, through which food processing methods and dietary combinations that tried to maintain nutrient adequacy and protect against overt malnutrition were progressively retained, even in contexts of limited food diversity and fluctuating resource availability. Techniques such as fermentation, soaking, thermal treatment, drying, and strategic food pairing were widely adopted across cultures [7,8,9] and were transmitted empirically, yet they frequently resulted in improved digestibility, enhanced mineral availability, and stabilization of labile micronutrients [10,11,12]. Traditional diets were shaped by necessity because explicit scientific understanding was lacking [13].

Food processing in ancestral foodways does not merely preserve raw materials but actively transforms their nutritional and functional properties. Structural modification of carbohydrates and proteins, reduction in antinutritional factors, microbial metabolism, and matrix interactions can profoundly influence nutrient bioaccessibility and downstream physiological effects [14,15]. When considered collectively at the dietary level, such transformations may contribute to dietary resilience—defined here as the capacity of a dietary pattern to maintain nutrient adequacy and metabolic stability in the face of environmental, physiological, or lifestyle stressors [16,17].

Recent interest in whole-food-based dietary patterns and minimally processed foods has renewed attention to the role of food structure and processing in nutrition [18,19]. However, existing research remains fragmented and often reductionist, focusing on isolated nutrients or individual foods rather than on the integrated pathways through which traditional diets operated. A framework-oriented synthesis that examines how traditional food practices may enhance nutrient bioavailability and functional interactions can therefore offer valuable insights for contemporary nutritional science. Accordingly, this review explores ancestral foodways as informal nutrient optimization strategies, emphasizing the nutritional mechanisms by which processing practices influence bioavailability, metabolic function, and dietary resilience. By integrating evidence from nutrition science, food chemistry, and human physiology, this review aims to derive transferable nutritional principles from traditional food systems that may inform contemporary dietary design and food system innovation.

## 2. Conceptual Framework: Traditional Diets as Informal Nutrient Optimization Architecture

In the context of this review, traditional food systems are defined as dietary infrastructures characterized by locally sourced raw materials, minimal (or nonexistent) industrial processing, and reliance on empirically transmitted food preparation and preservation practices developed prior to the widespread availability of modern food technologies [20,21,22]. These typically involve techniques such as fermentation, soaking, germination, thermal processing, and drying, applied at the household or community level and shaped by environmental, seasonal, and cultural constraints [23,24]. Importantly, the term “traditional” is used here to describe functional and technological characteristics rather than to imply nutritional superiority or cultural homogeneity. Traditional food systems encompass diverse practices across regions and historical periods, unified by their reliance on food processing methods that modify nutrient bioavailability and functional properties without external fortification or supplementation.

### 2.1. Nutrient Availability Beyond Nutrient Quantity

In conventional nutritional discourse, nutrient adequacy is often evaluated in terms of absolute intake relative to recommended dietary allowances. However, nutrient intake alone does not capture the complex processes that govern nutrient bioaccessibility, absorption, metabolism, and physiological utilization [25,26,27]. Nutrient enhancement, as used in this review, refers to the capacity of a dietary architecture to boost the effective availability and functional integration of nutrients within the human organism, rather than merely increasing their nominal content.

Key determinants of nutrient-enhanced accessibility include food structure [25], neutralisation of antinutritional factors, physicochemical interactions among nutrients [28], and the influence of processing on nutrient release and stability [12].

Ancestral dietary patterns also rely on consistent food-pairing structures that shape digestion and absorption at the meal level [29]. The habitual co-consumption of cereals with fermented or acidic foods, vegetables with lipid-containing components, or plant staples with fermented dairy creates biochemical environments that may favor nutrient solubilization, micellar formation, and enzymatic efficiency [30,31,32,33]. Rather than optimizing individual nutrients in isolation, these dietary patterns try to influence multiple nutritional dimensions simultaneously. This perspective reframes traditional diets not as static cultural practices, but as adaptive, maintaining nutritional adequacy through recurrent, framework-level strategies [34,35]. The evidence discussed in this review spans multiple levels, including mechanistic, in vitro, and human studies, which differ in strength and generalizability. Accordingly, the interpretation of these mechanisms within real-world dietary patterns should be considered context-dependent and, in some cases, indirect.

### 2.2. Dietary Resilience as an Emergent Nutritional Property

Dietary resilience is introduced here as an emergent property of nutrient-optimized food frameworks. It refers to the ability of a dietary pattern to maintain nutritional adequacy, metabolic stability, and functional flexibility under conditions of environmental variability, seasonal constraint, or physiological stress. Unlike sustainability, which focuses mainly on environmental outcomes, dietary resilience emphasizes the biological robustness of the diet–host interaction. In this context, dietary resilience is not equivalent to nutrient adequacy, metabolic stability, or sustainability, although it overlaps with these constructs. Rather, it is used here as an integrative concept referring to the capacity of a dietary framework to maintain nutrient utilization efficiency and physiological functionality under variable conditions. Nutrient adequacy reflects intake relative to requirements, metabolic stability refers to physiological responses such as glycemic control, and sustainability addresses environmental and system-level outcomes. Dietary resilience, as defined in this review, focuses specifically on the robustness of the diet–nutrient–host interaction. Traditional diets, shaped by repeated exposure to scarcity and variability, often display features consistent with dietary resilience. These include reliance on storable staples, diversification of nutrient sources through processing, and repeated use of techniques that preserve or enhance nutrient availability over time. Understanding dietary resilience from a nutritional perspective may offer insights into why certain traditional dietary patterns support metabolic health despite limited food diversity or the absence of modern fortification.

### 2.3. Aims and Scope

The aim of this article is to provide a conceptual synthesis of how traditional food systems can enhance nutrient bioavailability and utilization through processing methods, food pairing, and interactions within the food structural environment, to identify recurring mechanisms and derive transferable nutritional principles, which are synthesized in a dedicated section. The objective of this synthesis is therefore interpretative and hypothesis-generating, rather than to provide definitive evidence-based conclusions. Specifically, this work seeks to (i) identify recurring nutrient-related challenges inherent to plant- and mixed-based traditional diets; (ii) examine processing strategies such as fermentation, soaking, and extended cooking that mitigate nutrient trade-offs; and (iii) illustrate how representative dietary patterns—including Mediterranean, agrarian cereal–legume, and East Asian systems—embed these mechanisms within everyday dietary practices. By reframing traditional diets through a nutrient-centered lens, this article aims to contribute to a more integrated understanding of nutrient adequacy that extends beyond isolated nutrient intake toward framework-level nutritional resilience. To our knowledge, this is among the first attempts to systematically conceptualize traditional food systems as nutrient optimization architectures grounded in mechanistic evidence. Within this framework, dietary resilience is introduced as a central organizing concept linking processing, food structure, and nutrient utilization across dietary systems.

## 3. Conceptual Approach and Evidence Sources

### 3.1. Analytical Design

This article adopts a conceptual synthesis framework to examine traditional food systems as informal nutrient optimization strategies. The analysis focuses on identifying recurring and plausible mechanistic pathways through which food processing, food pairing, and structural properties of foods influence nutrient bioavailability, absorption, and metabolic responses. Rather than aiming for exhaustive coverage of all traditional diets, the objective is to extract transferable nutritional principles that emerge consistently across diverse dietary contexts

### 3.2. Literature Identification

Relevant literature was identified through targeted searches of PubMed, Scopus, and Web of Science, covering the period from 2000 to 2024, with inclusion of earlier seminal studies where mechanistically relevant. Studies were selected based on their relevance to the conceptual aims of the review, with priority given to human intervention studies and well-established mechanistic evidence. Observational and in vitro studies were included to support mechanistic interpretation where human data were limited. No formal inclusion/exclusion criteria or quality scoring system were applied, consistent with the narrative and hypothesis-generating nature of the work. When conflicting evidence was identified, findings were interpreted cautiously and used to highlight variability and context dependence rather than to derive definitive conclusions. Search terms included combinations of keywords such as traditional diets, fermentation, food processing, nutrient bioavailability, food structure, antinutritional factors, mineral absorption, and dietary patterns. Additional sources were identified through backward citation tracking of key reviews and primary studies.

### 3.3. Evidence Selection and Integration

Evidence was selected based on its relevance to mechanisms of nutrient bioaccessibility, absorption, and utilization in the context of traditional food practices. The synthesis integrates findings across study types to identify converging evidence on how processing techniques (e.g., fermentation, soaking, thermal treatment) and food combinations modify nutrient functionality within mixed meals (Table 1).

### 3.4. Scope and Limitations

This work does not aim to provide a systematic review, meta-analysis, or formal quality assessment of included studies. Instead, it offers a mechanistically oriented synthesis intended to generate hypotheses and highlight consistent nutritional patterns across traditional dietary systems. As such, the selection of evidence is not exhaustive, and interpretations are qualitative. The proposed framework should therefore be considered as a basis for further empirical validation rather than definitive evidence.

## 4. Traditional Food Processing Practices as Nutrient Optimization Strategies

**Figure 1:**
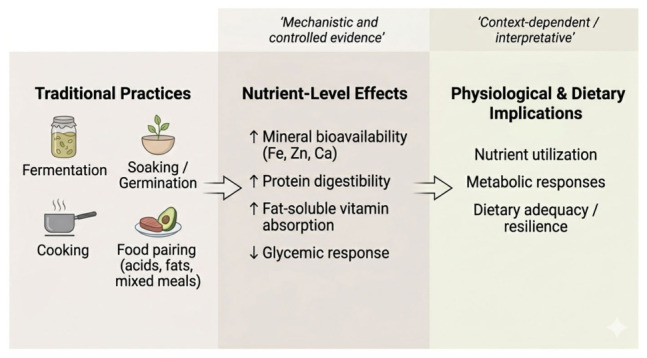
Traditional practices and nutritional effects.

Ancestral food frameworks rely on a limited set of processing techniques that modify the nutritional properties of raw foods. When applied repeatedly across staple foods, such techniques function as informal nutrient optimization strategies at the dietary level. The mechanisms described below derive from different levels of evidence, including controlled experimental studies and mechanistic research. Their interpretation at the level of whole dietary patterns is therefore inferential and should be understood as a conceptual integration rather than a direct demonstration of population-level effects (Figure 1).

### 4.1. Fermentation

Fermentation is one of the most influential traditional processing practices affecting nutrient availability and metabolic function. Through the activity of lactic acid bacteria, yeasts, and other microorganisms, it modifies carbohydrate and protein matrices, reduces antinutritional factors, and generates organic acids, peptides, vitamins, and microbial metabolites [37,38,39,40,75]. From a nutritional perspective, it enhances mineral bioavailability primarily by degrading phytates and lowering pH through organic acid production, thereby increasing the solubility and absorption of minerals such as iron, zinc, calcium, and magnesium [41]. Concurrently, microbial proteolysis improves protein digestibility and releases bioactive peptides that may exert physiological effects beyond basic nutrition [36]. Fermentation also stabilizes or increases the availability of certain B-group vitamins and produces metabolites that influence gut microbial ecology and host metabolic signaling [42]. Importantly, the nutritional impact of this process is not limited to the presence of live microorganisms. Non-viable microbial components and fermentation-derived metabolites can remain biologically active within the food microstructure, contributing to immunomodulatory, barrier-supportive, and metabolic effects [76,77]. These effects are mechanistically plausible, with emerging but still limited direct human evidence. These combined mechanisms position fermentation as a central mechanism through which traditional diets can enhance nutrient efficiency, not just quantity.

### 4.2. Soaking, Germination, and Enzymatic Activation

Soaking and germination represent another class of traditional practices that can improve nutrient availability, particularly in plant-based staple foods. These techniques activate endogenous enzymes, including phytases and amylases, which reduce antinutritional factors and modify carbohydrate structure [43,44,78]. Soaking grains, legumes, and seeds, traditionally done to improve taste and shorten thermal treatments, initiates phytate hydrolysis and mineral release, while germination further enhances this process by increasing enzymatic activity and modifying fiber composition [43]. Germination can also increase the content or bioaccessibility of certain vitamins, amino acids, and phenolic compounds [45,46]. From a metabolic standpoint, these processes reduce postprandial glycemic responses by altering starch structure and slowing carbohydrate digestion, an effect demonstrated in controlled settings, though effects depend on food composition and context. Unlike modern processing aimed at refining or fractionating foods, soaking and germination preserve the integrity of the food’s physicochemical environment while selectively improving nutrient accessibility [41]. When integrated into habitual dietary patterns, these practices are nutrient boosting by enhancing the efficiency of nutrient uptake from staple foods that otherwise contain poorly bioavailable micronutrients.

### 4.3. Thermal Processing and Combined Traditional Techniques

Thermal processing is universally present and plays a dual role. Heat treatment improves food safety, palatability, and digestibility, while also modifying the stability and bioaccessibility of nutrients [47]. Cooking denatures proteins, gelatinizes starches, and disrupts plant cell walls, thereby facilitating enzymatic digestion and nutrient release [48,49,50,51]. In parallel, thermal processing reduces naturally occurring toxins and antinutritional factors, including lectins and certain enzyme inhibitors [52,53]. Ancestral techniques frequently combine thermal processing with other techniques, such as fermentation or soaking, resulting in synergistic nutritional effects [79]. For example, heat treatment generally inactivates antinutritional compounds, and subsequent fermentation further boosts mineral solubility and generates bioactive metabolites [80]. Although some heat-sensitive vitamins and phytochemicals may be partially degraded [81,82], traditional practices often balance these losses by improving overall nutrient utilization and digestibility at the dietary level.

From a nutritional perspective, the relevance of thermal processing lies not in maximizing the retention of individual nutrients, but in optimizing the functional availability of nutrients within realistic dietary contexts.

### 4.4. Food Pairing, Functional Food Environment Effects, and Nutrient Synergy

Ancestral food frameworks are structured around recurring patterns of food pairing that shape nutrient bioavailability through food architecture interactions. These combinations influence digestion, absorption, and metabolic use of nutrients, often enhancing nutritional efficiency beyond what single foods could achieve alone [83]. Such effects are increasingly recognized in nutrition science but have long been embedded in traditional dietary practices.

A well-established example from controlled human studies is that of iron bioavailability. Non-heme iron from plant sources is poorly absorbed in isolation but is significantly enhanced when consumed alongside organic acids (e.g., lactic, acetic) or vitamin C–rich foods [84]. In traditional diets, fermented vegetables, sourdough breads, or acidic beverages are frequently consumed with cereal- or legume-based meals, creating a biochemical environment that promotes iron solubility and uptake [85]. Similarly, phytate degradation during fermentation, combined with organic acid production, further improves the bioavailability of iron, zinc, calcium, and magnesium from plant matrices [11,86,87].

Fat-soluble vitamin absorption represents another key food-structure-driven effect. Vitamins A, D, E, and K require dietary lipids for optimal micellar incorporation and intestinal uptake. Traditional meals commonly pair vegetables or fermented plant foods with dairy, eggs, or animal fats, facilitating the absorption of carotenoids and fat-soluble vitamins [88,89]. Fermented dairy products additionally contribute vitamin K_2_ (menaquinones), which interacts synergistically with calcium and vitamin D in bone and cardiovascular health, illustrating how food combinations shape nutrient functionality, added to quantity [54,55].

Food pairing also modulates glycemic and metabolic responses. The inclusion of protein-, fiber-, or fat-rich foods alongside carbohydrate staples slows gastric emptying, reduces postprandial glucose excursions, and improves insulin dynamics [56]. Fermented cereals, when consumed with dairy or vegetable-based accompaniments, exemplify this interaction by combining organic acids, bioactive peptides, and structural fibers within a mixed meal context, leading to more favorable metabolic outcomes than refined carbohydrate foods consumed alone [57]. Obviously, effects vary depending on dietary context and population.

Protein quality and amino acid availability are additional examples of nutrient synergy shaped by traditional food pairing. Plant-based protein sources often have limiting essential amino acid profiles (e.g., lysine in cereals) and reduced digestibility due to fiber, antinutritional factors, or protein structure. Traditional diets frequently may address these limitations through complementary protein combinations, such as cereals paired with legumes or dairy, which together ensure a more balanced amino acid profile and improved protein utilization [58]. Fermentation contributes to the enhancement of protein quality by partially hydrolyzing proteins into peptides and free amino acids, increasing digestibility and bioavailability [59]. For example, fermented dairy products supply highly digestible protein and bioactive peptides that, when consumed alongside cereal-based foods, improve overall amino acid adequacy of the meal [60]. Similarly, fermentation of cereals and legumes reduces protease inhibitors and modifies protein structure, facilitating enzymatic digestion [61,62]. These effects explain how the traditional food architecture optimizes protein utilization at the meal level, allowing diets with moderate total protein content to meet physiological requirements through improved efficiency even without increased intake.

Beyond host digestion, food combination contexts contribute to the extension of gut microbial metabolism. Diverse food associations provide a broader spectrum of fermentable substrates, micronutrients, and bioactive compounds that may support microbial cross-feeding and metabolic flexibility [90]. The co-delivery of fibers, polyphenols, organic acids, and microbial metabolites can enhance short-chain fatty acid production and microbial vitamin synthesis, indirectly influencing host nutrient status and metabolic regulation [91,92,93].

Taken together, these examples illustrate that traditional food pairing patterns function as nutrient synergy frameworks, where bioavailability, metabolic efficiency, and physiological impact are shaped by interactions between foods, not by individual nutrients in isolation. All these mechanisms may contribute to nutritional adequacy, although direct causal evidence at the dietary-pattern level remains limited. This perspective reinforces the need to evaluate nutrients within dietary matrices and meal patterns, aligning with contemporary calls in nutrition science to move beyond reductionist nutrient-centric models toward framework-based approaches.

### 4.5. Illustrative Examples from Traditional Dietary Patterns

Across multiple traditional dietary patterns, recurring food pairings exemplify these matrix-driven nutrient synergies. When considered within whole dietary models, these mechanisms may interact and contribute to observed nutritional outcomes; however, such extrapolations remain interpretative and context-dependent. The following dietary patterns are presented as illustrative examples of recurring nutritional mechanisms rather than as homogeneous or internally consistent systems. Considerable regional, cultural, and temporal variability exists within each of these dietary frameworks.

#### 4.5.1. Mediterranean-Type Diets

Mediterranean-type diets are some of the most extensively studied dietary patterns and are associated with reduced cardiometabolic risk, lower all-cause mortality, and improved metabolic health [63,64,65]. Beyond epidemiological associations, Mediterranean-type dietary patterns provide a useful model for examining how nutrient utilization may be shaped by recurring food combinations and processing practices, rather than by isolated nutrient intake alone.

These dietary patterns may reflect how nutrient adequacy emerges from repeated combinations of foods and processing practices, rather than from high intake of isolated nutrients. Their apparent resilience likely arises from multiple overlapping mechanisms—organic acid exposure, lipid-mediated absorption, fermentation, and mineral–fiber interactions—that collectively may improve nutrient utilization. This perspective reinforces the need to evaluate nutrients within dietary structures rather than in isolation.

From a micronutrient perspective, Mediterranean-type diets appear to support the bioavailability of iron, zinc [66], and fat-soluble vitamins, despite a relatively high reliance on plant-based foods. Non-heme iron absorption may be facilitated by the frequent co-consumption of organic acids and polyphenol-rich vegetables alongside iron-containing foods [67]. The habitual use of acidic components such as tomatoes, fermented vegetables, and wine-derived organic acids may lower gastric pH and promote iron solubilization, partially offsetting the inhibitory effects of phytates present in legumes and whole grains. Human absorption studies suggest higher non-heme iron bioavailability in mixed Mediterranean-style meals compared with isolated plant-based meals of similar iron content [94,95,96]. Fat-soluble vitamin absorption represents another feature influenced by dietary structure. The consistent use of olive oil as a culinary fat may enhance intestinal absorption of carotenoids (β-carotene, lycopene) and vitamins A, D, and K through micelle formation [97]. Intervention studies have shown higher plasma carotenoid concentrations when vegetables are consumed with added fats, although these effects remain context-dependent [98,99]. Fermented dairy products, such as yogurt and traditional cheeses, contribute additional nutrient-level effects. These foods provide calcium, bioavailable protein, and vitamin K_2_ (menaquinones [100]), while fermentation reduces lactose content and modifies protein structure, improving digestibility. The co-consumption of fermented dairy with plant foods may further enhance mineral absorption through lactic acid–mediated reductions in intestinal pH. Observational and intervention studies have reported associations between fermented dairy intake within Mediterranean-type dietary patterns and improved bone and metabolic markers [101,102], although disentangling individual nutrient contributions remains challenging. Taken together, these recurring food combinations suggest that Mediterranean-type dietary patterns may function as nutrient-distribution frameworks, in which absorption efficiency and metabolic handling of micronutrients are shaped by dietary structure. However, the extent to which these mechanisms translate into consistent population-level effects remains variable and influenced by broader dietary and lifestyle factors.

#### 4.5.2. Traditional Agrarian Cereal–Legume Diets as Mineral Bioavailability Modulators

Traditional agrarian diets, historically widespread across large parts of Europe, South and East Asia, the Middle East, and sub-Saharan Africa, are characterized by a high reliance on cereals and legumes as primary energy and protein sources [103,104]. The long-term nutritional viability of these somewhat heterogeneous dietary infrastructures therefore depends not on nutrient content alone, but on processing and preparation strategies that modify mineral accessibility. From a nutritional standpoint, such diets might pose an inherent challenge: although rich in minerals, cereals and legumes also contain substantial levels of phytates and polyphenols that limit mineral bioavailability.

Fermentation, soaking, germination, and extended cooking are recurrent features of traditional agrarian food preparation and exert measurable effects on mineral bioavailability [14,68]. In certain European and West Asian contexts, sourdough leavening of wheat and rye activates endogenous and microbial phytases that degrade phytic acid, releasing bound iron, zinc, and calcium [69]. Controlled feeding studies demonstrate that mineral absorption from fermented cereal products is significantly higher than from non-fermented equivalents, even when total mineral content is comparable, with particularly pronounced effects in long-fermented whole-grain products.

Comparable strategies are observed in agrarian dietary frameworks of Africa and South Asia, where cereals and legumes are frequently subjected to soaking, spontaneous fermentation, or mixed fermentation–cooking processes. Fermented cereal foods and legume-based preparations common in these regions exhibit reduced phytate content and improved mineral solubility relative to unprocessed staples, supporting micronutrient adequacy in settings with limited access to animal-source foods [70,86].

Food pairing further modulates nutrient utilization in agrarian-type diets. The habitual co-consumption of cereals with acidic foods—such as fermented vegetables, sour milk, or vinegar-based preparations—reduces luminal pH and enhances iron solubility. Legume-based dishes consumed alongside allium vegetables or fermented components may similarly mitigate inhibitory effects on mineral absorption. Such combinations recur across agrarian-type cuisines and may function as implicit compensatory mechanisms, allowing predominantly plant-based diets to meet micronutrient requirements without reliance on animal foods. From a metabolic perspective, leavening-induced changes in starch structure and organic acid content also influence glycemic responses. Long-leavened cereal products exhibit lower postprandial glucose excursions than rapidly fermented or industrially processed breads, contributing to improved glucose homeostasis [71]. Together, these findings might suggest that agrarian diets operate as nutrient-adaptive, processing, pairing, and transforming mineral-dense but poorly bioavailable foods into nutritionally functional staples.

This model illustrates how traditional plant-based diets might remain nutritionally viable not through nutrient density alone, but through processing-driven modulation of bioavailability and postprandial metabolism.

#### 4.5.3. East Asian Dietary Patterns

In the maritime East Asian regions, particularly Japan and coastal areas of Korea and China, regular consumption of seaweeds (e.g., kombu, wakame, nori) is integrated with fermented soy products, rice, and vegetables. Fermented soy foods contribute to bioavailable protein, peptides, and isoflavones, while the process reduces antinutritional factors such as phytates, thereby improving the bioavailability of minerals, including iron, zinc, and calcium [72]. The inclusion of seaweeds supplies iodine and additional trace minerals, supporting thyroid function and micronutrient adequacy in populations with limited dairy intake. Fiber-rich vegetables and fermented components modulate postprandial glycemic responses by slowing carbohydrate absorption and influencing gut microbial metabolism, leading to improved glucose homeostasis. Together, these dietary combinations may reflect how microbial fermentation, mineral provision, and carbohydrate quality interact to shape metabolic outcomes beyond total nutrient intake.

In contrast, pastoral and agro-pastoral-type dietary systems incorporating fermented dairy are more prevalent in continental East Asia, including Mongolia, Inner Mongolia, parts of western and northern China, and Central Asian-influenced regions, where fermented milk products are traditionally consumed alongside cereals or plant foods. Fermentation of milk generates organic acids, bioactive peptides [73], and microbial metabolites that improve protein digestibility and facilitate mineral absorption, particularly calcium and magnesium [74]. The presence of dietary fats within fermented dairy matrices further supports the absorption of fat-soluble vitamins, including vitamins A, D, and K_2_, an effect supported by human studies, although the magnitude varies with meal composition. When consumed alongside cereals or plant foods, organic acids and fermentation-derived compounds reduce the inhibitory effects of phytates and improve overall mineral bioavailability [11,105]. These interactions suggest the manner in which pastoral-type dietary patterns optimize nutrient utilization through the combined effects of fermentation, lipid-mediated absorption, and structure-mediated modifications of plant-derived foods.

Across these patterns, fermentation functions as a pre-digestive nutritional technology, reshaping micronutrient availability and metabolic responses before ingestion more than relying on post-absorptive compensation [33].

#### 4.5.4. Blue Zone Dietary Patterns as Convergent Nutrient Optimization Frameworks

Dietary patterns observed in so-called Blue Zone regions can be interpreted not merely as longevity-associated diets, but as long-standing, population-scale nutritional systems that have suggested ecological and physiological durability over generations [106,107]. While these populations are often cited just in relation to longevity outcomes, the present interpretation is exploratory and does not imply a causal or uniform relationship between dietary structure and health outcomes. Although culturally distinct, these dietary models share structural characteristics consistent with enhanced nutrient utilization. In Ikaria and Sardinia, Mediterranean-type patterns integrate legumes, vegetables, whole grains, olive oil, and fermented dairy, supporting mineral bioavailability and lipid-mediated absorption of fat-soluble micronutrients. The traditional Okinawan pattern emphasizes sweet potatoes, vegetables, and fermented soy foods, combining high fiber density with fermentation-modified plant proteins and organic acids that influence glycemic responses and mineral accessibility [108]. The Nicoyan maize–bean framework [109,110] incorporates preparation methods that reduce phytate content and improve mineral bioavailability within a predominantly plant-based structure. Across these diverse contexts, recurring reliance on fermentation, complementary protein pairing, minimally refined staples, and matrix-based meal composition may suggest convergent evolution toward nutrient-efficient dietary architectures. In this sense, Blue Zone-type dietary systems function as real-world models of long-term nutritional resilience rather than as prescriptive longevity formulas [111].

### 4.6. Trade-Offs, Constraints, and Limitations of Traditional Processing

While traditional food processing practices often contribute to the enhancement of nutrient bioavailability and functional properties, they also involve trade-offs and constraints that must be critically acknowledged. Certain techniques, such as salting [112] or prolonged fermentation, may increase sodium content or generate undesirable metabolites if poorly controlled [33,113,114]. Thermal processing can reduce the content of heat-labile vitamins, and spontaneous fermentation may introduce variability in nutrient composition and safety [24,115]. Moreover, these pathways evolved in response to specific environmental and cultural contexts and may not uniformly meet modern nutritional needs [116]. Energy scarcity, limited protein availability, or imbalanced micronutrient profiles were not uncommon historically, and traditional diets should not be idealized as nutritionally complete in all circumstances.

Recognizing these limitations is essential for translating traditional principles into contemporary nutrition. Replicating traditional diets wholesale is impossible, but modern applications should selectively adopt processing strategies that may enhance nutrient efficiency while mitigating potential risks. This critical perspective reinforces the value of traditional food configurations as sources of nutritional insight rather than as prescriptive models.

## 5. Nutrient-Level Implications of Traditional Food System Structures

The mechanisms described in the preceding sections—processing, food pairing, and matrix interactions—have direct implications for nutrient bioavailability, utilization, and metabolic pathways. This section synthesizes their consequences across major nutrient domains, highlighting how traditional dietary structures influence nutritional outcomes at the nutrient level.

### 5.1. Minerals

Traditional food systems address mineral bioavailability constraints inherent to plant-based foods [27]. Fermentation, soaking, and extended cooking reduce phytate and oxalate content, thereby increasing the bioaccessibility of iron, zinc, calcium, and magnesium. When combined with habitual consumption of organic acids from fermented foods or acidic plant components, these processes enhance mineral solubility and intestinal absorption. The process is supported by mechanistic and controlled studies, with variable confirmation in human settings. As a result, diets with moderate total mineral content may meet physiological requirements through improved absorption efficiency.

### 5.2. Protein and Amino Acids

Protein adequacy in traditional diets is shaped by both processing and food pairing. Fermentation improves protein digestibility by partial proteolysis and reduction in protease inhibitors, while complementary combinations of plant and animal proteins may enhance essential amino acid balance at the meal level. These strategies allow diets with moderate protein density to achieve sufficient amino acid availability, illustrating how protein quality and utilization can be augmented without reliance on high absolute protein intake.

### 5.3. Vitamins, Lipids, and Lipid-Associated Components

Traditional dietary structures influence both the stability and absorption of vitamins. Fermentation can preserve or increase the availability of certain B-group vitamins and vitamin K_2_ [10], while lipid-containing food matrices facilitate the absorption of fat-soluble vitamins and carotenoids. The recurrent pairing of vegetables with dietary fats and fermented dairy supports efficient vitamin uptake, reinforcing the importance of dietary context over isolated vitamin content. In addition to previously discussed mechanisms, dietary lipids and lipid-associated compounds represent an important, yet variably expressed, component of traditional food systems. The quantity and quality of dietary fats differ substantially across traditional dietary patterns, influencing both nutrient absorption and physiological effects. Traditional diets often include sources of long-chain polyunsaturated fatty acids, such as arachidonic acid from terrestrial animal foods and long-chain omega-3 fatty acids from marine environments, which contribute to membrane function and metabolic regulation [117]. Furthermore, lipid presence within meals is essential for the absorption of fat-soluble vitamins (A, D, E, and K) and carotenoids, with traditional dietary structures frequently facilitating this through the co-consumption of plant foods with animal fats or vegetable oils [118,119]. Tocopherols, carotenoids, and vitamin D status are therefore shaped not only by intake but by dietary context and lipid availability [120,121]. In parallel, traditional diets are often rich in polyphenol-containing foods, including vegetables, legumes, fruits, and fermented products, which may interact with nutrient absorption and metabolic pathways. While the bioavailability of polyphenols is complex and context-dependent, their integration within mixed meals and traditional processing practices may contribute to broader metabolic effects [122,123]. These aspects further support the concept that nutrient functionality in traditional diets is determined by integrated dietary structures rather than isolated components.

### 5.4. Carbohydrates and Glycemic Regulation

Processing-induced modifications of carbohydrate structure, particularly through leavening, influence postprandial glycemic responses [57]. Organic acids, altered starch fractions, and food structure complexity slow carbohydrate digestion and absorption, contributing to lower glycemic excursions. When carbohydrate staples are consumed within mixed meals containing proteins, fats, and fibers, these effects are further amplified, supporting glycemic stability without elimination of carbohydrate-rich foods.

### 5.5. Integrated Nutrient Utilization

Across nutrient classes, the defining feature of traditional food systems is not maximization of individual nutrients but coordinated enhancement of nutrient utilization. By embedding processing and pairing strategies into everyday dietary practices, these frameworks reduce nutrient losses, mitigate antinutritional effects, and improve metabolic efficiency. This integrated approach allows nutritional adequacy to be maintained under conditions of limited food diversity, seasonal variability, and modest energy intake. Importantly, these structural features of the ancestral food architecture manifest in measurable physiological domains, from fractional nutrient absorption to postprandial metabolic responses and circulating biomarkers of nutrient status.

## 6. Implications for Contemporary Nutrition and Public Health

While the preceding sections detail specific mechanisms and dietary examples, this section synthesizes their broader implications for nutrient-focused dietary design and contemporary nutrition challenges. Some degree of conceptual reiteration is retained across sections to maintain continuity between mechanistic explanations, dietary examples, and their broader nutritional implications, while minimizing redundancy where possible. Based on the recurring mechanisms identified across traditional dietary systems, several transferable principles of nutrient optimization can be derived (Figure 1).

The evidence synthesized in this review supports a reframing of traditional food systems as functional nutritional architectures and not only as collections of culturally specific foods. Their relevance lies not in nostalgia or heritage but in their ability to contribute to the nutrient intake adequacy through structural dietary features that enhance bioavailability, metabolic efficiency, and physiological utilization under constrained conditions. From a scientific perspective, they challenge reductionist models that prioritize isolated nutrient intake over dietary context [124].

Traditional processing and food-pairing practices demonstrate that nutrient effectiveness is contingent on structural food context interactions, meal composition, and metabolic handling. Ignoring these dimensions risks underestimating the nutritional value of diets that are moderate in nutrient density but optimized for utilization. This has direct implications for how nutrient requirements, dietary reference intakes, and food-based guidelines are interpreted and applied. In particular, reliance on intake-based adequacy models that do not account for matrix-dependent bioavailability may overestimate requirements in structurally optimized dietary contexts or underestimate risk in poorly structured but quantitatively sufficient diets. Integrating utilization efficiency into dietary assessment frameworks could refine interpretations of micronutrient adequacy, especially in plant-forward dietary patterns where absorption dynamics are highly context-dependent.

In public health and policy contexts, ancestral food configurations may offer pragmatic insights for addressing micronutrient inadequacy without reliance on fortification or supplementation alone. Processing-driven improvements in mineral and vitamin bioavailability, coupled with glycemic modulation and enhanced protein digestibility, suggest that dietary structure itself can function as a low-cost nutritional intervention. The dietary structure can mitigate nutritional risk even where food diversity or purchasing power is limited [125]. Such principles are particularly relevant for aging populations, low-resource settings, and sustainability-oriented dietary transitions.

**Figure 2:**
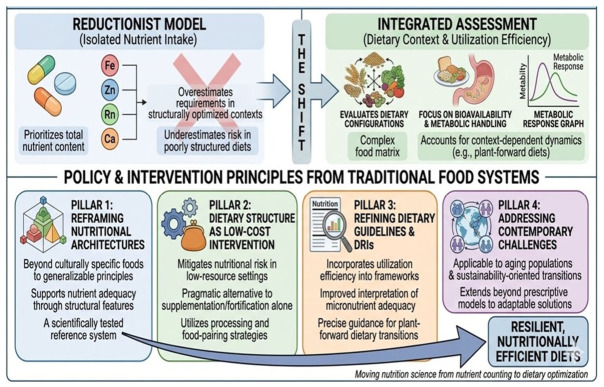
Implications for contemporary nutrition and public health.

Finally, these findings underscore the need for nutrition research frameworks that move beyond single-nutrient endpoints toward integrated assessments of dietary configurations. Evaluating foods and diets based on utilization efficiency, structural interactions, and postprandial metabolic responses may provide a more accurate foundation for both research and policy than nutrient content alone. Ancestral food frameworks thus serve not as prescriptive models, but as empirically tested reference systems for designing resilient, nutritionally efficient diets in contemporary contexts. These considerations provide the basis for the transferable principles outlined below (Figure 2).

## 7. Transferable Nutrient Optimization Principles

**Figure 3:**
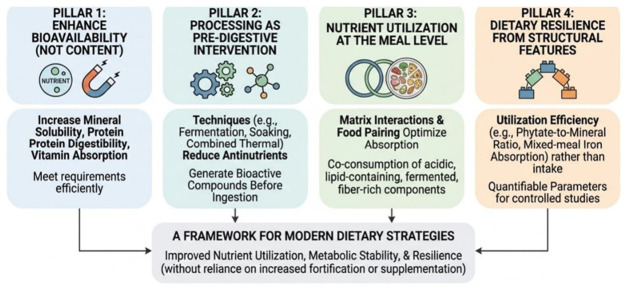
Transferable nutrient optimization principles: from traditional to contemporary nutrition.

Building on this conceptual definition, dietary resilience can be tentatively operationalized through measurable indicators related to nutrient utilization efficiency. Based on the mechanisms discussed above, the following principles can be derived (Figure 3):

Nutrient adequacy depends on bioavailability rather than nutrient content alone. Traditional food settings employ processing and pairing strategies that increase mineral solubility, protein digestibility, and vitamin absorption, allowing diets with moderate nutrient density to meet physiological requirements efficiently.

Food processing functions as a pre-digestive nutritional intervention. Techniques such as fermentation, soaking, germination, and combined thermal processing systematically reduce antinutritional factors, modify macronutrient structure, and generate bioactive compounds before ingestion, thereby reducing reliance on post-absorptive compensation mechanisms.

Nutrient utilization is shaped at the meal level through matrix interactions and food pairing, and not at the level of isolated foods. The habitual co-consumption of acidic, lipid-containing, fermented, and fiber-rich components may create biochemical environments that enhance mineral absorption, fat-soluble vitamin uptake, amino acid availability, and glycemic stability.

Finally, dietary resilience emerges from structural features. To enhance empirical applicability, dietary resilience can be operationalized through measurable indicators reflecting nutrient utilization efficiency rather than intake alone. Such indicators may include phytate-to-mineral molar ratios as predictors of mineral bioavailability, mixed-meal fractional iron absorption capacity, fat-mediated postprandial carotenoid response, matrix-sensitive protein digestibility indices, and attenuation of postprandial glycemic excursions within whole-meal contexts. Framing resilience in terms of these quantifiable parameters allows traditional nutrient optimization strategies to be evaluated within controlled human studies and compared with modern dietary models. Traditional food systems are consistent with maintaining nutritional functionality under conditions of limited food choice, seasonal variability, and modest energy intake by embedding these nutrient-optimizing strategies into everyday practices.

Together, these principles provide a framework for translating insights from traditional food systems into modern dietary strategies aimed at improving nutrient utilization, metabolic stability, and resilience without reliance on increased fortification or supplementation.

## 8. Limitations and Future Directions

This conceptual synthesis has several limitations that should be acknowledged. First, the evidence based on traditional food systems and nutrient-boosting techniques is inherently heterogeneous. Many studies rely on compositional analyses, in vitro digestion models, or animal experiments, while high-quality human intervention trials remain relatively scarce. As a result, causal links between traditional processing practices, nutrient bioavailability, and long-term health outcomes cannot always be firmly established.

Second, these food frameworks are characterized by substantial variability in raw materials, processing conditions, and cultural practices. Fermentation time, microbial consortia, heat exposure, and food combinations differ across regions and households, limiting standardization and comparability across studies. This variability complicates extrapolation of findings to contemporary, industrialized diets.

Third, much of the available literature examines individual nutrients or isolated bioactive compounds, whereas traditional diets function through complex food matrices and habitual dietary patterns. The synergistic effects of nutrient–nutrient interactions, food pairing, and processing-induced food structure modifications are still insufficiently captured by reductionist study designs.

Future research should therefore prioritize well-designed human feeding studies in order to assess nutrient bioavailability and metabolic responses to traditionally processed foods within realistic dietary contexts. Integrated approaches combining food composition analysis, digestion models, microbiome profiling, and nutritional biomarkers can better characterize matrix effects. Also, comparative studies evaluating traditional processing techniques alongside modern food technologies can identify transferable strategies for improving nutrient efficiency in contemporary diets. Greater attention has to be paid to under-studied micronutrients (e.g., iodine, iron, zinc, vitamin K_2_, B-vitamins) whose bioavailability is strongly influenced by processing and food combinations. Such work would help bridge the gap between traditional knowledge and evidence-based nutritional recommendations.

## 9. Conclusions

We propose a nutrient-centered reinterpretation of traditional food systems as adaptive dietary frameworks that may enhance nutrient bioavailability and utilization through processing, food pairing, and food environment interactions. The interpretations presented should be understood as conceptually derived and hypothesis-generating, particularly where direct comparative human evidence remains limited. Across diverse cultural contexts, they embed strategies that may mitigate nutrient trade-offs, improve metabolic efficiency, and sustain nutritional adequacy under conditions of limited food diversity and variable resource availability. The evidence reviewed indicates that nutritional resilience in traditional diets does not arise from exceptional nutrient density or uniform food composition, but from recurrent structural features that optimize the physiological use of nutrients. Fermentation, complementary food combinations, and within-food structural environment-driven effects act in concert to improve mineral absorption, protein quality, vitamin uptake, and glycemic regulation, highlighting the limitations of evaluating nutrients outside their dietary context. By reframing traditional food systems through the lens of nutrient utilization rather than cultural specificity, this work contributes to a broader understanding of how dietary structure is able to influence nutritional outcomes. These insights support a shift in nutrition research and guidance toward integrative approaches that account for bioavailability, metabolic handling, and meal composition alongside nutrient intake. Traditional diets appear to integrate these mechanisms in ways that may support nutritional adequacy, by no means infallible, but inspirational for designing nutritionally efficient, resilient diets suited to contemporary health and sustainability challenges.

## Figures and Tables

**Table 1:** Conceptual domains, mechanisms, and representative evidence.

Conceptual Domain	Mechanism/Process	Nutritional Effect	Type of Evidence	Representative References
**Food structure**	Food microstructure; physicochemical interactions within the matrix	Modulation of nutrient bioaccessibility and absorption	Mechanistic/in vitro/human	[25,26,27,28,29]
**Antinutritional factor reduction**	Phytate degradation; enzyme activation	Increased mineral bioavailability (Fe, Zn, Ca, Mg)	Human/in vitro	[24,27,36]
**Fermentation**	Microbial metabolism; organic acid production; proteolysis	↑ Mineral solubility; ↑ protein digestibility; production of bioactive metabolites	Human/experimental	[36,37,38,39,40,41,42]
**Soaking & germination**	Activation of endogenous enzymes (phytases, amylases)	↓ Antinutrients; ↑ nutrient accessibility; improved glycemic response	Experimental/human	[43,44,45,46,47]
**Thermal processing**	Protein denaturation; starch gelatinization; cell wall disruption	↑ Digestibility; ↓ antinutritional factors and inhibitors	Experimental/human	[29,47,48,49,50,51,52,53]
**Food pairing**	Organic acids, lipids, mixed-meal interactions	↑ Iron absorption; ↑ fat-soluble vitamin uptake; ↓ glycemic response	Human studies	[30,31,32,33,34,54,55,56,57,58]
**Protein complementation**	Cereal–legume and plant–animal protein combinations	Improved amino acid profile and protein utilization	Human/mechanistic	[58,59,60,61,62]
**Dietary patterns**	Integration of processing and food pairing at dietary level	System-level nutrient efficiency; metabolic stability; dietary resilience	Observational/intervention	[63,64,65,66,67,68,69,70,71,72,73,74]

## Data Availability

The original contributions presented in this study are included in the article. Further inquiries can be directed to the corresponding author.
